# Data on litterfall production and meteorology at an old-growth tropical dry forest in northwestern Mexico

**DOI:** 10.1016/j.dib.2020.105723

**Published:** 2020-05-19

**Authors:** Ana M. Velez-Ruiz, Lucia Nevescanin-Moreno, Martha L. Vargas-Terminel, Ana R. Flores-Espinoza, Juan C. Álvarez-Yépiz, Enrico A. Yépez

**Affiliations:** Departamento de Ciencias del Agua y Medio Ambiente, Instituto Tecnológico de Sonora, 5 de Febrero 818 sur, Col. Centro, Cd. Obregón, Sonora, México C.P. 85000

**Keywords:** Ecology, Litterfall, Seasonally dry ecosystems, Alamos Sonora

## Abstract

Chronological measurements of litterfall production can be used for understanding ecosystem dynamics such as net primary production and carbon cycling in highly seasonal ecosystems such as tropical dry forests (TDF). This paper presents data on litterfall production and meteorology in an old-growth TDF. The data was generated within the Monte Mojino Reserve located in the Sierra de Alamos – Rio Cuchujaqui Natural Protected Area in northwestern México. For litterfall collection, twenty randomly placed litterfall traps were installed to collect monthly litterfall production across four full growing seasons (48 monthly collections). Meteorological data were obtained from an automatic micrometeorological station that recorded data in situ from January 2013 to March 2019. The database includes litterfall production [g m^−2^ month^−1^], monthly rainfall [mm], air temperature [°C], relative humidity [%] and photosynthetic active radiation [µmol m^−2^ s^−1^].

Specifications Table

**Table:** 

Subject	Ecology
Specific subject area	Ecosystems ecology
Type of data	Tables Figures
How data were acquired	We generated litterfall data collecting litter from twenty 0.5 m-diameter litterfall traps made of synthetic mesh suspended at 75 cm from the ground. All traps were visited and cleaned every month between May and March of the following year. The meteorological data was acquired with the following instruments: rain gauge (TE525-L, Texas Electronics, Dallas, Texas, USA), a temperature and relative humidity probe (HMP45, Vaisala Inc., Helsinki, Finland) and a quantum Sensor (LI-COR, Lincoln, Nebraska, USA) to sense photosynthetic active radiation. All sensors were connected to a datalogger (CR3000, Campbell Scientific, Logan, Utah, USA). Litterfall samples were oven-dried for 48 hours at 65°C to obtain their dry weight in grams. Meteorological measurements were collected every 30 minutes and averaged to obtain the monthly records of each variable.
Data format	Raw Analyzed
Parameters for data collection	Data were collected within an old-growth tropical dry forest in a protected natural reserve. Due to remote accesses litterfall bags were cleaned once every month. Due to lack of funding and personnel it was not possible to collect litterfall samples during 2014 and 2017. Micrometeorological sensors were appropriately calibrated and the use of proper enclosures to avoid weathering were utilized.
Description of data collection	Litterfall samples we collected manually in the field and processed in the lab to obtain dry weights, meteorological data was collected automatically in dataloggers that were interrogated monthly.
Data source location	Institution: Instituto Tecnologico de Sonora City/Town/Region: Monte Mojino Reserve, Alamos, Sonora Country: Mexico. Latitude and longitude (and GPS coordinates, if possible) for collected samples/data: N 26.996830, W -108.789190
Data accessibility	With the article

## Value of the Data

• Chronological measurements of litterfall production can be used for understanding ecosystem dynamics such as net primary production and carbon cycling in highly seasonal ecosystems such as tropical dry forests.
• Intra- and interannual patterns of litterfall production can serve as a proxy for phenological changes in ecosystems to describe environmental controls on ecosystem processes.
• Understanding environmental controls on litter production allow the generation and calibration of ecosystem process models and validation of remote sensing products.
• Data on litterfall production across time can be used by scientists interested in comparing litterfall production across the continental distribution of the tropical dry forest in the Americas.
• Measurements of litter production should be valuable for long term ecological monitoring efforts in natural protected areas.

## 1 Data Description

The dataset includes 48 collections of monthly litterfall production across four full growing seasons between May and March of years 2013-2014, 2015-2016, 2016-2017 and 2018-2019, and a continuous time series of meteorological data from January 2013 to March 2019. Litterfall data were collected in an old-growth tropical dry forest, a highly seasonal ecosystem in the foothills of the Sierra Madre in northwestern Mexico [1]. The litterfall data included four full growing seasons that show the strong effects of the rainfall associated with the North American monsoon on the productivity of dry ecosystems.

Meteorological data were collected with an automated system, mounted at 15 m height (about 2 meters above canopy). Environmental variables were automatically sampled every minute, and averaged to 30-minute periods by the datalogger. We then calculated the monthly amounts of precipitation [mm] and monthly means of air temperature [°C], relative humidity [%] and photosynthetic active radiation [µmol m^−2^ s^−1^].

These data were collected to understand ecosystem phenological changes and dynamics of net primary production and how these are related to changes in environmental conditions.

The dataset content is described in section 1.1, variation of monthly litterfall production is presented in section 1.2. In section 1.3 is presented the meteorological data.

**Fig. 1:**
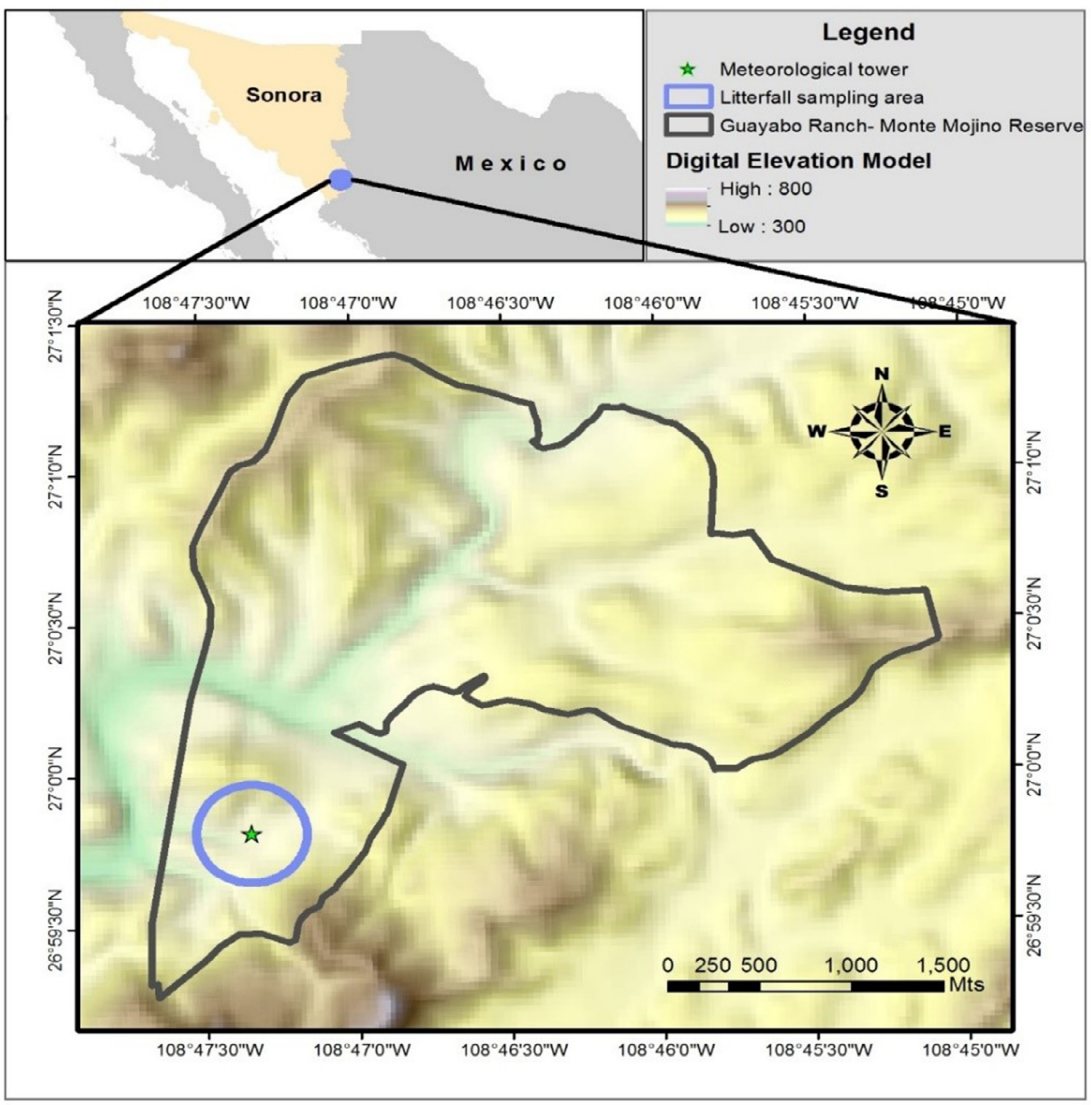
Location map of the study site: Old-growth tropical dry forest at Rancho "El Guayabo", in the Monte Mojino Reserve, located in the municipality of Alamos, state of Sonora, Mexico. Circle delimits the sampling area where the litterfall traps were located the star symbolizes the micrometeorological station.

The litterfall data lacks information in the 2014-2015 and the 2017-2018 growing seasons Fig. 1.

### 1.1 Dataset content

Table 1.

**Table 1:** Description of the dataset.

Column Name	Description	Units
Site	Study Site: Álamos, Sonora, México.	Text
Successional Stage	Successional forest state: Old-growth Forest	Text
Year	Collection year.	Text
Month	Collection month.	Text
Litterfall Production	Monthly litterfall production.	Number [g m^−2^ month^−1^]
Rainfall	Monthly rainfall amount.	Number [mm]
Air Temperature	Monthly mean air temperature record.	Number [°C]
Relative Humidity	Monthly mean relative humidity record.	Number [%]
PAR	Monthly mean photosynthetic active radiation record.	Number [µmol m^−2^s^−1^]

### 1.2 Litterfall production

Fig. 2.

**Fig. 2:**
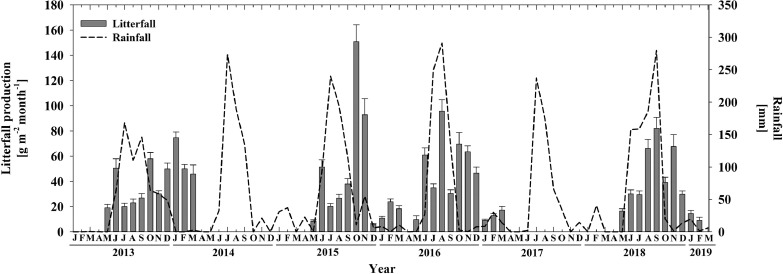
Time series data of monthly litterfall production [g m^−2^ month^−1^] and rainfall [mm] through the period May 2013 – March 2019 in an old-growth tropical dry forest in northwestern Mexico. Error bars indicate the standard deviation of 20 litterfall traps (n=20). No field sampling was carried out in 2014 and 2017.

### 1.3 Meteorological data

Fig. 3.

**Fig. 3:**
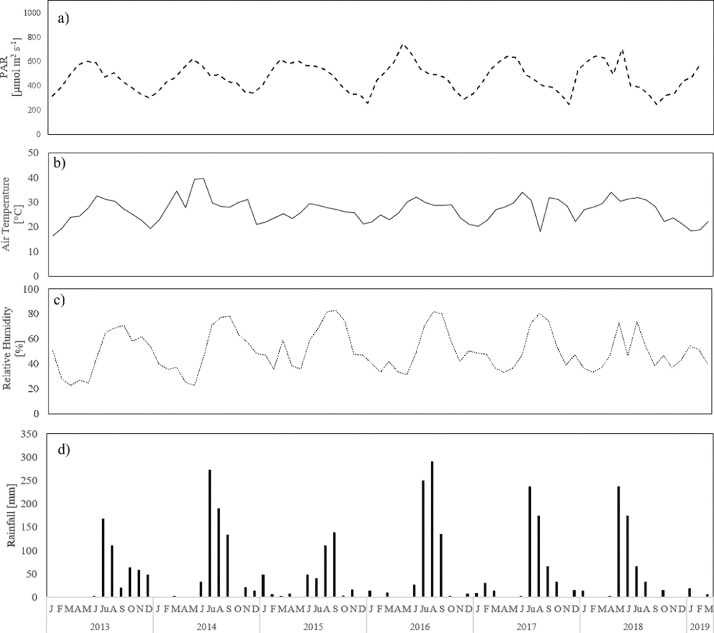
Times series of meteorological data from January 2013 to March 2019: a) photosynthetic active radiation (PAR) [µmol m^−2^ s^−1^], b) air temperature [°C], c) relative humidity [%] and d) rainfall [mm].

## 2 Experimental design, materials and methods

Data on monthly litterfall production was acquired by installing twenty traps, randomly distributed across a study site with an area of 28.35 hectares. Traps were made of synthetic mesh with a diameter of 0.5 m and suspended 75 cm from the ground. Samples were collected by hand and placed in paper bags each month. Once in the laboratory, we removed insects and branches over 2 cm in diameter, and then placed the samples in an oven for 48 hours at 65°C, to obtain their dry mass in grams using a digital scale. To calculate the amount of litterfall per unit area, the litterfall weight of each sample was divided by the area of the trap (0.19 m^2^), expressing the values in grams of litter per square meter per month (g m^−2^ month^−1^). This methodology is comparable to the one used by Anaya et al. (2012) [2].

Data on meteorological variables was obtained using a rain gauge (Texas Electronics, Dallas, Texas, USA), a temperature and relative humidity sensor (HMP45, Vaisala Inc., Helsinki, Finland.) and a quantum sensor (LI-190SB-L, LI-COR, Lincoln, Nebraska, USA) that were connected to a datalogger (CR3000, Campbell Scientific, Logan, Utah, USA.), which interrogated sensors every minute and recorded means in hard media every 30 minutes. From half-hour means we calculated the monthly amount rainfall [mm], and monthly averages of air temperature [°C], relative humidity [%] and photosynthetic active radiation [µmol m^−2^ s^−1^].

## Declaration of Competing Interest

The authors declare that they have no known competing financial interests or personal relationships which have, or could be perceived to have, influenced the work reported in this article.

## Appendix Supplementary materials

Image, application 1

Image, application 2
